# Supplementary material for: Microbial Signature in Adipose Tissue of Crohn’s Disease Patients
Source: J Clin Med. 2020 Jul 31;9(8):2448. doi: 10.3390/jcm9082448 (PMC7465250; doi:10.3390/jcm9082448)
Supplement: Supplementary file 1 [file jcm-09-02448-s001.pdf]

A.

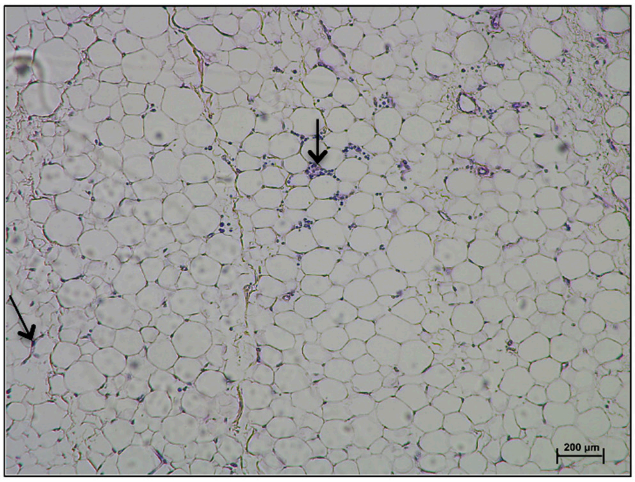

B.

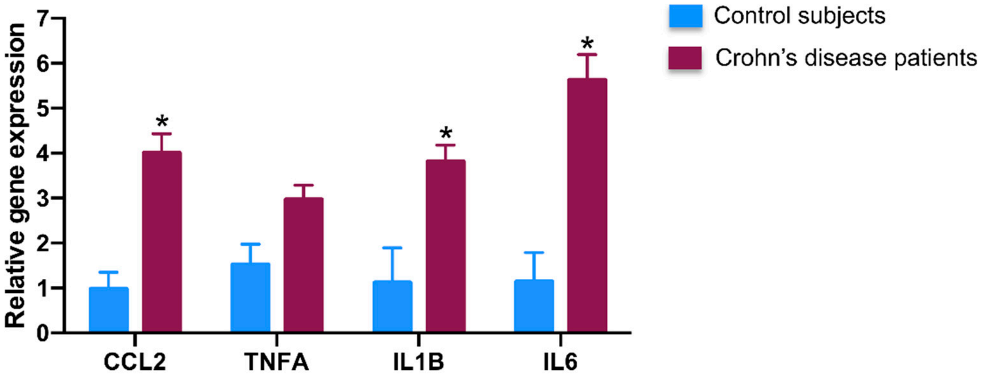

Supplementary Figure 1. (A) H&E staining of thin sections of creeping fat adipose tissue from one representative patient with Crohn's disease. Magnification is 200 micrometres in the insets. Arrows are used to indicate inflammatory cells in insets. **(B)** Gene expression of inflammatory markers in the creeping fat adipose tissue of Crohn's disease patients versus adipose tissue of control subjects. N= 8 per group *$p<0.05$ versus control group. No parametric test (U-Mann Whitney) was used.

Abbreviations: H&E, haematoxylin & eosin; CCL2, chemokine (C-C motif) ligand 2; TNFA, tumor necrosis factor alpha; IL1B, Interleukin 1 Beta; IL6, Interleukin 6.
